# Intravenous Administration of Manuka Honey Inhibits Tumor Growth and Improves Host Survival When Used in Combination with Chemotherapy in a Melanoma Mouse Model

**DOI:** 10.1371/journal.pone.0055993

**Published:** 2013-02-07

**Authors:** Maria J. Fernandez-Cabezudo, Rkia El-Kharrag, Fawaz Torab, Ghada Bashir, Junu A. George, Hakam El-Taji, Basel K. al-Ramadi

**Affiliations:** 1 Department of Biochemistry, College of Medicine and Health Sciences, UAE University, Al Ain, United Arab Emirates; 2 Department of Medical Microbiology & Immunology, College of Medicine and Health Sciences, UAE University, Al Ain, United Arab Emirates; 3 Department of Surgery, College of Medicine and Health Sciences, UAE University, Al Ain, United Arab Emirates; 4 Department of Surgery, Tawam Hospital –Johns Hopkins Medicine, Al Ain, United Arab Emirates; IDI, Istituto Dermopatico dell'Immacolata, Italy

## Abstract

Manuka honey has been recognized for its anti-bacterial and wound-healing activity but its potential antitumor effect is poorly studied despite the fact that it contains many antioxidant compounds. In this study, we investigated the antiproliferative activity of manuka honey on three different cancer cell lines, murine melanoma (B16.F1) and colorectal carcinoma (CT26) as well as human breast cancer (MCF-7) cells in vitro. The data demonstrate that manuka honey has potent anti-proliferative effect on all three cancer cell lines in a time- and dose-dependent manner, being effective at concentrations as low as 0.6% (w/v). This effect is mediated via the activation of a caspase 9-dependent apoptotic pathway, leading to the induction of caspase 3, reduced Bcl-2 expression, DNA fragmentation and cell death. Combination treatment of cancer cells with manuka and paclitaxel *in vitro*, however, revealed no evidence of a synergistic action on cancer cell proliferation. Furthermore, we utilized an *in vivo* syngeneic mouse melanoma model to assess the potential effect of intravenously-administered manuka honey, alone or in combination with paclitaxel, on the growth of established tumors. Our findings indicate that systemic administration of manuka honey was not associated with any alterations in haematological or clinical chemistry values in serum of treated mice, demonstrating its safety profile. Treatment with manuka honey alone resulted in about 33% inhibition of tumor growth, which correlated with histologically observable increase in tumor apoptosis. Although better control of tumor growth was observed in animals treated with paclitaxel alone or in combination with manuka honey (61% inhibition), a dramatic improvement in host survival was seen in the co-treatment group. This highlights a potentially novel role for manuka honey in alleviating chemotherapy-induced toxicity.

## Introduction

Honey has been used for more than 2000 years as traditional medicine in different cultures, particularly for its wound healing properties. The antimicrobial properties of honey have been well described in the literature. Intrinsic properties of honey like high osmolarity and acidity, as well as the presence of flavonoids and phenolic acids are responsible for its antibacterial and antioxidant activities [1]. In addition to its antimicrobial, antioxidant and tissue-protective activities, recent reports have highlighted multiple roles for honey in enhancing immune responses, including the induction of inflammatory cytokine production by macrophages [2], stimulation of neutrophil migration [3] and enhanced antibody production [4]. Whether the multitude of honey activities is mediated by the same or different active fractions remains to be fully elucidated.

Manuka honey, obtained from nectar collected by honey bees (*Apis Mellifera*) from the New Zealand manuka tree (*Leptospermum scoparium*), is a complex mixture of carbohydrates, fatty acids, proteins, vitamins and minerals containing various kinds of phytochemicals with high phenolic and flavonoid content [5]. While manuka honey shares constituents, e.g. glucose-oxidases, with other honeys it also contains other phytochemical factors that potentiate its antibacterial activity like methylglyoxal [6]. This gave rise to a classification system adopted for active manuka honey, known as unique manuka factor (UMF), an indication of its antibacterial activity [7]. Previous studies addressing the mechanisms of the anti-bacterial activity of manuka honey identified a number of potential active constituents, including several phenolic compounds that act as scavengers of superoxide anion radicals [8]–[10]. There is evidence that antibacterial activity of manuka is independent of its role in inducing inflammatory cytokines during innate immune responses. A 5.8 kD, heat-sensitive, protease-resistant, component, that was devoid of any antibacterial activity was identified to be responsible for the induction of cytokine production via interaction with TLR4 on macrophages [11]. These studies suggest the presence of unique, yet-to-be-characterized, constituents with desired activities in manuka honey. However, an investigation of the anti-proliferative properties of manuka honey has not been undertaken.

Perhaps, one of the oldest known uses for honey is in wound healing. There is extensive scientific and clinical evidence to support the utilization of honey for wounds, skin reactions and damage to epithelial barriers following radiotherapy and chemotherapy [12]. In patients with chronic wounds or burns, honey has been shown to stimulate angiogenesis and epithelialization, promoting more efficient healing [13], [14]. More recently, several reports demonstrated that honey, being rich in polyphenols and flavonoids, has anti-proliferative effects against cancer cells [3], [15]–[17]. However, the mechanisms for the anti-cancer effect are still to be fully elucidated. The current study is aimed at investigating the effect of manuka honey on the growth of cancer cells, using both *in vitro* as well as *in vivo* approaches. Our findings provide mechanistic evidence for the induction of apoptosis in cancer cells by manuka treatment and further highlight a novel role for systemically-administered manuka as both an anti-cancer agent and an adjuvant in combination with standard chemotherapeutic agents.

## Materials and Methods

### Ethics Statement

All animal studies were carried out in accordance with, and after approval of, the Animal Research Ethics Committee of the College of Medicine and Health Sciences, UAE University (protocol# AE/03/35).

### Cell lines and mice

The murine melanoma B16.F1 (H-2^b^) and human breast cancer cell line MCF-7 were generously provided by Dr Salem Chouaib (Institut Gustave Roussy, Villejuif, France) [18]. The mouse colon carcinoma cell line CT26 (H-2^d^) was a kind gift from Dr Siegfried Weiss (Helmholtz Centre for Infection Research, Braunschweig, Germany). Tumor cells were maintained in DMEM supplemented with 10% FCS, L-glutamine, sodium pyruvate, essential amino acids, non-essential amino acids, pen/strep, gentamicin, and 2-ME (all reagents from GIBCO-Invitrogen, Paisley, UK). C57BL/6 mice were obtained from Harlan Olac (Bicester, U.K.) and bred in the animal facility of the College of Medicine and Health Sciences, UAE University. For the present studies, male mice were used in experiments at 8–12 weeks of age. Animals were housed in groups of five in plastic cages with a controlled light and dark cycle of 12 h each at 24–26°C. They were maintained on standard laboratory animal diet with food and water ad libitum.

### Reagents

Paclitaxel, hereafter referred to as taxol, (Sigma, St. Louis, MO, USA), was diluted in sterile saline solution, divided into aliquots and stored at −80°C. Before each experiment, the drug was further diluted to the desired final concentration for i.v. administration or freshly diluted in culture medium for *in vitro* studies. Manuka honey (UMF® 10+, Honeyland NZ Ltd, New Zealand) was diluted in sterile saline or culture medium for *in vivo* or *in vitro* use, respectively, following aseptic procedures throughout. Manuka concentrations are expressed as % w/v. All preparations of manuka were freshly prepared on the day of use.

### In vitro Viability Assay

Tumor cells were seeded into 96-well plate at 5×10^3^ cells/well in supplemented DMEM culture medium. Serial dilutions of manuka (range 0.3% to 5%) prepared in sterile culture medium were then added to each well. As positive controls, cells were treated with taxol at 10 ng/ml or 50 ng/ml (equivalent to 11.7 or 58.5 nM, respectively) final concentration. In some experiments, tumor cells were treated with a combination of manuka and taxol added at the start of culture, as indicated in the figure. All determinations were done in duplicate. After 24, 48 or 72 hr incubation at 37°C, cell viability was determined using CellTiter-Glo® Luminescent cell viability assay (Promega, Madison, WI, USA). Luminescent signal was measured using Glomax Luminometer system. Data were presented as percent cell viability of experimental groups compared with that of the untreated cells, the viability of which is taken as 100%.

### Caspase Assays

Caspase-3/7 and caspase-8 activity were assayed using Caspase-Glo 3/7® and Caspase-Glo 8® assay kits, respectively (Promega). Briefly, B16.F1 cells were seeded (5×10^3^ cells/well) in 96- well plates and treated with Manuka (5% final concentration) or taxol (10 ng/ml or 50 ng/ml) for 24 hrs. Subsequently, 100 µl of a cell lysis solution containing a luciferase substrate derivative, Ultra-Glo™ Recombinant Luciferase and Mg^2+^ were added and cells incubated for 2 hrs at room temperature. Luminescence was measured using a Glomax luminometer. Duplicate plates were also set-up for determination of cell viability. The luminescent signal was then normalized to control, as per manufacturer's recommendation, and adjusted relative to extent of viable cell number. The data is reported as relative fold increase compared to control, non-treated, cells. All determinations were carried out in duplicate for each group.

### Flow Cytometric Analysis

B16.F1 melanoma cancer cells were cultured in 6-well plates at a concentration of 2×10^5^ cells/well with different concentrations of manuka (0.3% to 5.0%) or taxol (10 ng/ml). After a 24 hr-incubation, cells were collected, washed and stained with Annexin V-FITC apoptosis detection kit (BD Pharmingen, CA, USA) following manufacturer protocol, and analyzed on a FACSCalibur (BD Biosciences, San Jose, CA, USA).

### Western Blot Analysis

B16.F1 melanoma cells (5×10^5^ cells/well) were cultured in the presence of different concentrations of manuka (0.6%, 1.25%, 2.5%, 5%) or taxol (50 ng/ml) for 24 hrs or 72 hrs in a 6 well plate in supplemented DMEM medium. At the end of the incubation period, cells were harvested, washed and lysed, as previously described [19], [20]. Briefly, cell extracts were prepared in lysis buffer containing protease inhibitors PMSF (1 mM), aprotinin (5 µg/ml), pepstatin (1 µg/ml), and leupeptin (1 µg/ml). The lysates were kept on ice for 30 min and vigorously vortex mixed and sonicated (3 pulses of 3 seconds each) before centrifugation at 15,000 *g* for 10 min. Aliquots (100 µg) of total proteins were resolved on 10% SDS-PAGE and then transferred to a nitrocellulose membrane. The membrane was incubated with anti-PARP (1∶1000; Cell Signaling), anti-caspase 9 (1∶1000; Cell Signaling) or anti-Bcl2 (1∶500, Santa Cruz Biotechnology) rabbit polyclonal antibody overnight. After thorough washing in TBST, membranes were incubated for 1 hr with appropriate HRP-conjugated goat anti rabbit secondary antibody and developed using enhanced chemiluminescence detection system (Pierce, Rockford, IL, USA).

### DNA Fragmentation assay

B16.F1 cells were seeded at a density of 2×10^5^ cells/well in a 6 well plate containing DMEM with 5% FBS, and treated for 72 hrs with indicated concentration of manuka and taxol, as previously described. Cells were then lysed in Lysis buffer (10 mM Tris-HCl, pH 7.4, 10 mM EDTA and 0.2% Triton X-100) and partially degraded DNA was isolated and purified by centrifugation and phenol-chloroform extraction. Electrophoresis of DNA was performed on 1.5% agarose gel in TBE buffer at 50 V for 2 hrs. The gel was stained with ethidium bromide and the banding pattern visualized with a transilluminator.

### In vivo Toxicity Studies

C57BL/6 mice were injected intravenously with manuka honey (50% w/v) or saline two times a week for 3 weeks. At the end of the third week of treatment, mice were sacrificed under ether anesthesia. Blood was collected by venipuncture of the inferior vena cava into EDTA-containing tubes and cell counts of the total WBC, neutrophils, lymphocytes, RBC, platelets and monocytes were determined by Cell-Dyn Sapphire hematology analyzer (Abbott Diagnostics Division, Santa Clara, CA, USA). Chemistry analysis of the blood including creatinine, BUN, glucose, ALT, AST and LDH was performed using a Synchron clinical system (Beckman coulter, Fullerton, CA, USA). For all in vivo studies, mice were monitored on a daily basis for signs of morbidity (rapid weight loss, dehydration, slow movement). Moribund mice were routinely euthanized by CO_2_ asphyxiation.

### In vivo Tumor studies

The procedure for implanting B16.F1 tumor cells in syngeneic C57Bl/6 mice has been described [21], [22]. Briefly, 8-week-old mice were implanted s.c. with 2×10^5^ B16.F1 cells and staged to day 11. Tumor growth was followed by quantitative determination twice per week of the volume of tumor tissue, measured as the product of the perpendicular diameters using digital calipers, according to the formula: volume = LxW^2^/2. At day 11 post implantation, mice bearing B16.F1 tumors were given intravenous doses (100 µl per injection) of saline (control), 50% manuka suspension in sterile saline, taxol (10 mg/kg) or 50% manuka+taxol (10 mg/kg). All treatments were routinely administered twice per week, using freshly prepared reagents. Tumor growth and animal survival were followed for the subsequent 3–4 weeks. Mice were humanely sacrificed when tumor volume exceeded 8 cm^3^.

### Histology and Immunohistochemistry of Tumor Tissue

At days 20–24 post-treatment, tumors were resected and fixed in 10% formaline, dehydrated through graded ethanol, and embedded in paraffin. Paraffin-embedded tumors were sectioned serially at 25 µm intervals of 5 µm thickness using a rotary Shandon AS325 microtome (Shandon Lipshaw, Pittsburg, PA, USA). Sections were stained with haematoxylin and eosin (H&E), dehydrated in ascending concentrations of ethanol, cleared in xylene and mounted in DPX (Panreac, Barcelona, Spain). Apoptotic cells were detected by SignalStain® Cleaved Caspase-3 IHC detection kit (Cell Signaling Technology, Beverly, MA, USA) following manufacturer protocol. Images were captured using Olympus BX51TF microscope equipped with digital camera DP72 (Tokyo, Japan).

### Statistical Analysis

Statistical significance between control and treated groups was analyzed using the unpaired, two-tailed Student's t-test, using the statistical program of GraphPad Prism software (San Diego, CA). Survival analysis was performed by Kaplan-Meier survival curves and log-rank test, using the same GraphPad Prism program. Differences between experimental groups were considered significant when *P* values were <0.05.

## Results

Initial experiments were done to study the physiochemical characteristics of the manuka honey. Different dilutions of manuka were prepared directly in tissue culture medium in which the B16.F1 melanoma cells are routinely cultured and tested for their pH and osmolarity. The studies revealed that manuka solutions of concentrations up to 5% (w/v) were physiological (data not shown). Thus, all subsequent *in vitro* studies were carried out using manuka concentrations in the range of 0.3% to 5%.

### Manuka inhibits growth of cancer cells

**Figure 1:**
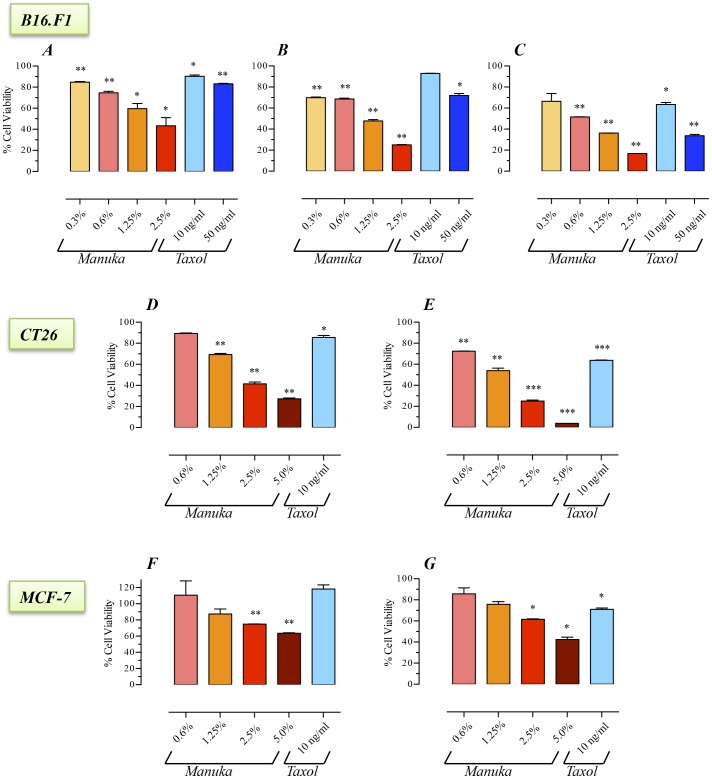
Inhibition of cancer cell proliferation by manuka honey. B16.F1 (graphs ***A–C***), CT26 (graphs ***D*** *,* ***E***) and MCF-7 (graphs ***F*** *,* ***G***) cells were plated at 5×10^3^ cells per well and incubated for 24 hr (graphs **A**, **D**, **F**), 48 hr (graph ***B***) or 72 hr (graphs ***C*** *,* ***E*** *,* ***G***) in the absence or presence of the indicated concentrations of manuka honey (range 0.3% to 5.0% w/v), or taxol (10 ng/ml or 50 ng/ml final concentration). At the end of the incubation period, cell viability was determined using CellTiter-Glo luminescent assay. Results are expressed as percentage viability in treated cell cultures compared to control, untreated, cells and are representative of 3 (for B16.F1 cells) or 2 (for CT26 and MCF-7 cells) independent experiments. Asterisks denote statistically significant differences in viability of experimental groups compared to control (*, *p*<0.05; **, *p*<0.01; ***, *p*<0.001).

The potential effect of manuka on cancer cell proliferation was investigated using three tumor cell lines differing in type and origin, the murine melanoma (B16.F1) and colon carcinoma (CT26) cells and the human breast cancer (MCF-7) cell line. Cells were incubated with different concentrations of manuka (range 0.3 to 2.5%) for 24–72 hrs. As a positive control, cells were cultured with taxol at a final concentration of 10 or 50 ng/ml. As shown in Figure 1, the addition of as little as 0.3% manuka to cells in culture resulted in a significant decrease in the viability of B16.F1 cells (panels *A–C*). This inhibitory effect on cell viability was dependent on both manuka concentration and total incubation time. By as early as 24 hrs, the viabilities of B16.F1 cells cultured with manuka at final concentrations of 0.3, 0.6, 1.25 and 2.5% were 85%, 75%, 60% and 43% of control (no manuka) cultures, respectively (Fig. 1A). In contrast, over the same time period, the viabilities of B16.F1 cells cultured in presence of 10 ng/ml or 50 ng/ml of the antineoplastic drug taxol were reduced to 90% or 83% of control, respectively (Fig. 1A). The decreased cell viability was more pronounced as the time of culture increased to 48 hrs (Fig. 1B) or 72 hrs (Fig. 1C). At the latter time point, cell viability was reduced to 17% in cell cultures treated with 2.5% manuka (Fig. 1C). Under the same conditions, cells cultured with 10 ng/ml or 50 ng/ml taxol had a reduction in viability to 64% or 34% of control, respectively. Essentially similar results were also observed with the CT26 (panels *D–E*) and MCF-7 (panels *F–G*) cancer cell lines. These results demonstrate that *in vitro* treatment of cancer cells with low concentrations of manuka resulted in significant inhibition of cell proliferation.

**Figure 2:**
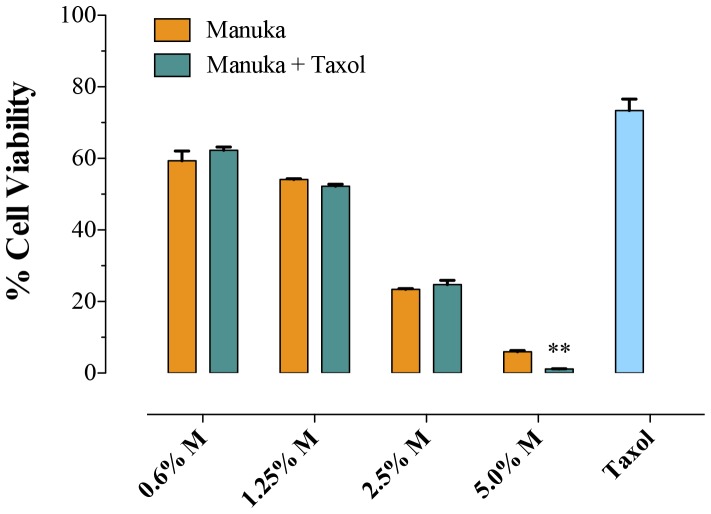
Co-treatment with manuka and taxol results in additive effect. B16.F1 cells were seeded at 1×10^3^ cells per well in a 96-well plate and incubated with the indicated concentrations of manuka, alone or in combination with taxol (10 ng/ml), for 72 hrs. Cell viability was determined using CellTiter-Glo luminescent assay. Results are expressed as percentage viability in treated cell cultures compared to untreated cells and are representative of 3 independent experiments. Asterisks denote statistically significant differences between corresponding cell cultures treated with each manuka concentration in absence or presence of taxol (*, *p*<0.05).

Next, experiments were undertaken to investigate the effect of co-treatment with manuka and taxol on proliferation of B16.F1 melanoma cells. Taxol (10 ng/ml) plus different concentrations of manuka (0.6% to 5.0%) were added simultaneously at the initiation of B16.F1 cell culture and cell viability was assessed 72 hrs later. As can be seen in Figure 2, increasing concentrations of manuka resulted in correspondingly lower cell viability, reaching a mean of 6% at the highest concentration of manuka used. Importantly, however, the extent of cell death achieved by a combination of taxol and manuka was essentially the same as seen with manuka alone (Fig. 2). This suggests that taxol and manuka act in an additive, not synergistic, manner when used in combination with melanoma cancer cells.

### Manuka induces apoptosis in cancer cells

**Figure 3:**
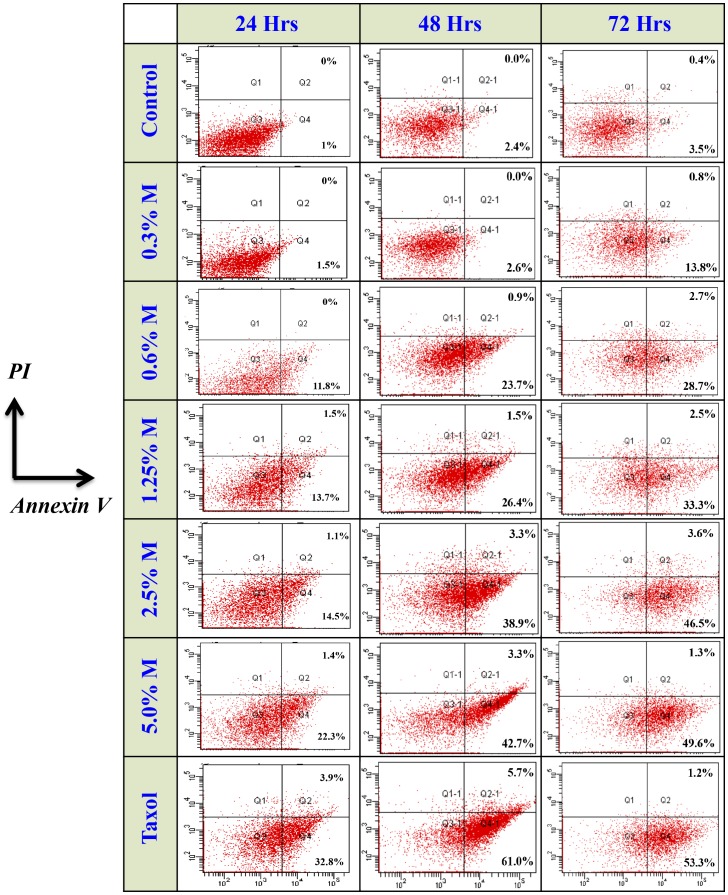
Manuka honey induces apoptosis in a dose-dependent manner. B16.F1 cells were treated for 24 hrs (left column), 48 hrs (center column) or 72 hrs (right column) with varying concentrations of manuka (M; range 0.3%–5.0%), taxol (10 ng/ml) or medium as control. At the end of the incubation period, cells were harvested and stained with Annexin V and PI, and analyzed by flowcytometry. The percentages of cells in early (Annexin V^+^, PI^−^; lower right quadrant) and late apoptotic-necrotic stages (Annexin V^+^, PI^+^; upper right quadrant) are shown. The results are representati**v**e of three independent experiments.

In the next series of experiments, we addressed the potential mechanism by which manuka was causing decreased cell viability. Loss of cell membrane asymmetry, detectable by Annexin V staining, represents one of the earliest events in apoptosis. B16.F1 cells were harvested at 24, 48, or 72 hrs after treatment with different concentrations of manuka honey (range 0.3% to 5.0%) or taxol (at a final concentration of 10 ng/ml), stained with Annexin V-FITC and PI, and subjected to flowcytometric analysis. As can be seen in Figure 3, there was a dose-dependent, and time-dependent, increase in the number of cells undergoing apoptosis (Annexin V-positive) after culture with increasing concentrations of manuka honey. At 24 hr post treatment, while the percent of Annexin V-positive cells in untreated control cultures was 1.0%, there were 1.5%, 11.8%, 13.7%, 14.5% and 22.3% apoptotic cells after culture with 0.3%, 0.6%, 1.25%, 2.5% and 5.0% manuka honey solution, respectively (left panels). In contrast, cells treated with taxol alone showed 32.8% apoptotic cells. Furthermore, in some cultures, a minor population of cells was observed to be positive for both Annexin V-FITC and PI, representing late apoptotic cells. These cells amounted to 1.0–1.5% of total in cell cultures treated with manuka (at 0.6% concentration or higher) and 3.9% in cells treated with taxol. The results of cell analysis following similar treatments for 48 and 72 hrs (center and right panels, respectively) demonstrate a similar dose-dependent trend in apoptosis, with the overall levels of apoptotic cells being higher than those observed at 24 hrs. For example, the percentage of total apoptotic cells following treatment with 0.3% manuka was 1.5%, 2.6% and 13.8% after 24, 48, and 72 hrs, respectively. The corresponding ratios of cell death following treatment with 1.25% manuka suspension were 15.2%, 27.9% and 35.8%, respectively. These findings suggest that the death of cancer cells following exposure to low concentrations of manuka honey occurs via an apoptotic mechanism.

**Figure 4:**
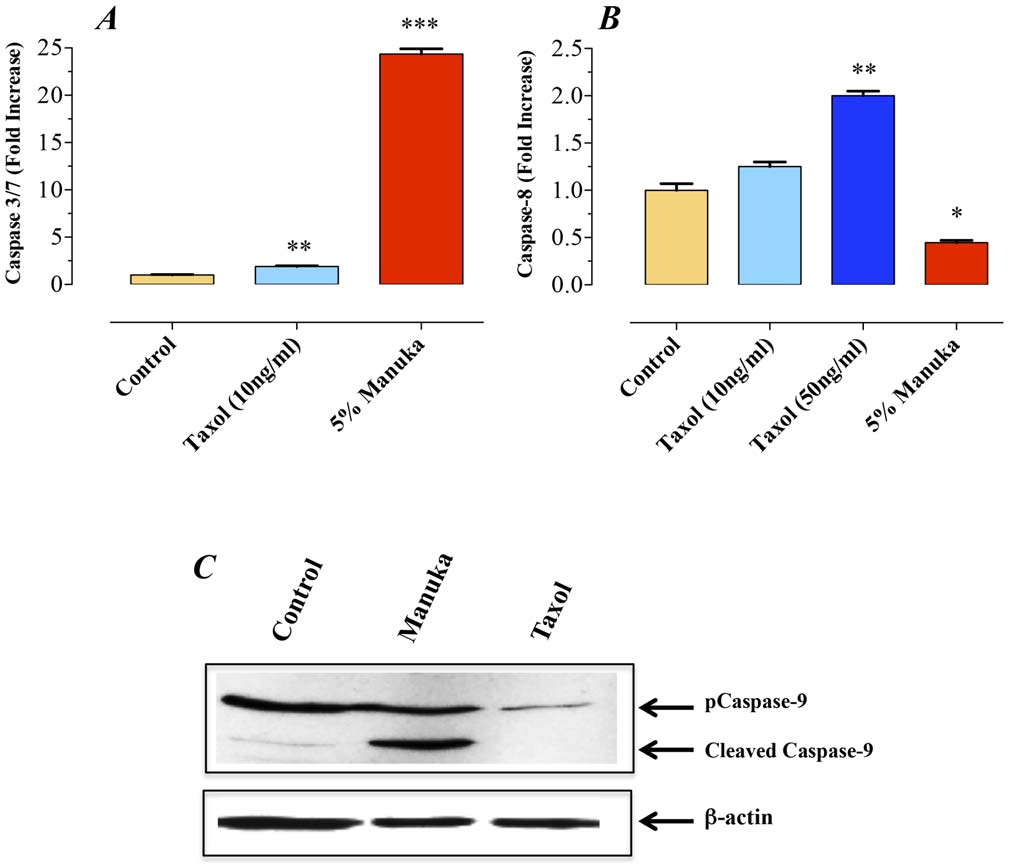
Manuka induces caspase-mediated apoptosis in cancer cells. B16.F1 melanoma cells were treated with manuka (5% w/v), taxol (10 or 50 ng/ml) or medium as control. After 24 hrs of culture, enzymatic activity of caspase 3/7 (graph ***A***) and caspase 8 (graph ***B***) were determined using specific kits and following manufacturer's recommendation. The data is presented as fold increase in caspase activity after normalization to the number of viable cells per culture. ***C***. Western blot analysis of caspase-9 activation B16.F1 cells treated with manuka or taxol. Whole cell extracts were prepared after a 24-hr treatment with manuka (5% w/v) or taxol (10 ng/ml). Protein extracts were resolved on 10% SDS-PAGE and immunoblotted with caspase-9-specific ployclonal antibody capable of detecting both full length and cleaved forms of caspase-9. The cell extracts were also probed with an antibody against β-actin as a control for protein loading.

A critical component for the initiation of the apoptosis pathway is the sequential recruitment of a number of caspases leading to the activation of the effector caspase-3. This, in turn, leads to the cleavage of a number of vital cellular substrates required for cell viability [23]. We next explored the mechanism of apoptosis induction in manuka-treated cancer cells. B16.F1 melanoma cells exposed to manuka (5% final concentration) for 24 hrs exhibited a 24-fold increase in caspase 3/7 activity (Fig. 4A). The induction of caspase 3/7 activity in manuka-treated cells was mainly due to activation of caspase-9 (Fig. 4C) but not caspase-8 (Fig. 4B). In sharp contrast, treatment of the cells with taxol (10 ng/ml) led to a 2-fold increase in caspase 3/7 activity (Fig. 4A) and this was associated with a 2-fold increase in caspase-8 activity (Fig. 4B). No evidence for induction of caspase-9 was observed in taxol-treated cancer cells (Fig. 4C). This implies that taxol-induced cell death occurs mainly via the extrinsic pathway, which is in agreement with previous observations [24]. These findings demonstrate that manuka activates caspase-dependent apoptosis in cancer cells, a process initiated through caspase-9, implicating the intrinsic pathway in manuka-induced cell death.

**Figure 5:**
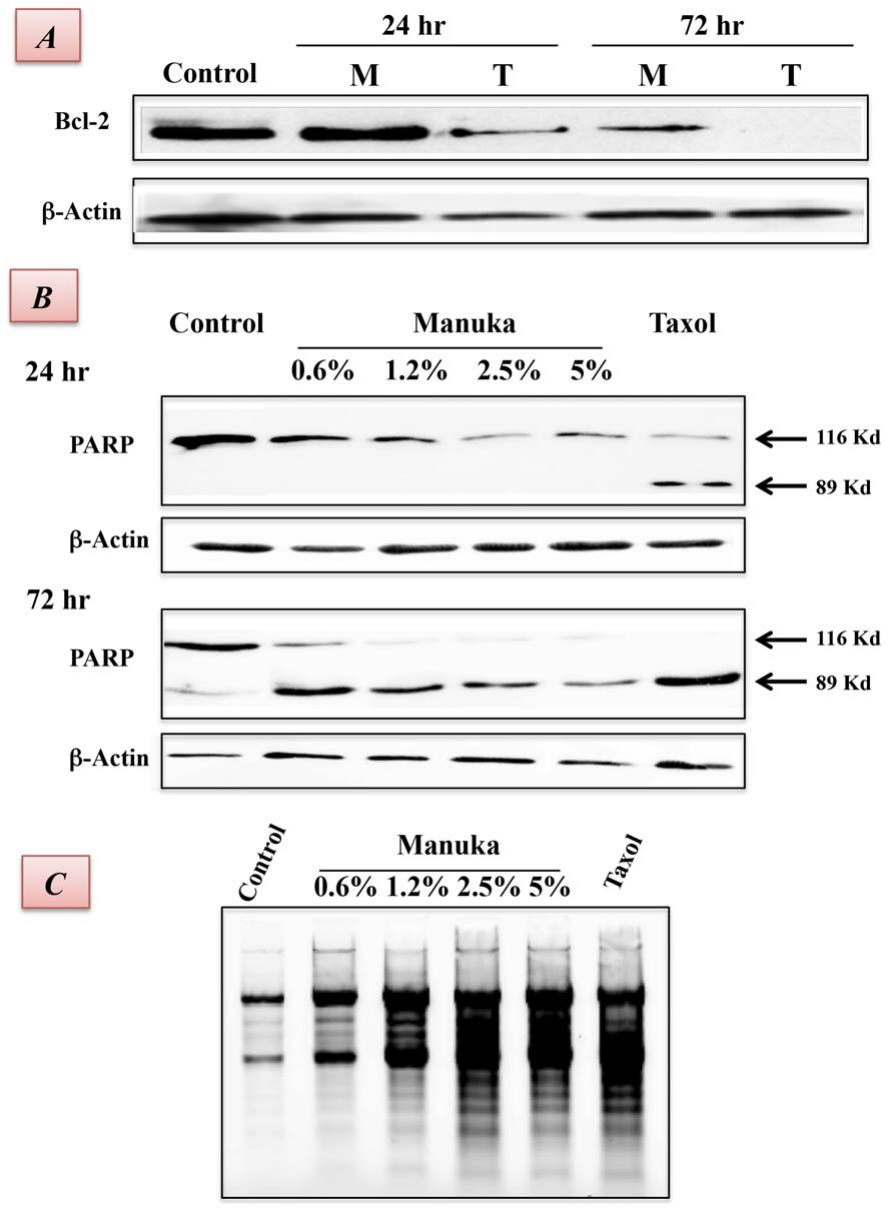
Evidence for late apoptotic events induced by manuka honey in cancer cells. ***A***. B16.F1 cells were incubated for 24 hrs or 72 hrs in the absence or presence of Manuka (*M*; 5%) or taxol (*T*; 50 ng/ml). Whole cell extracts (100 µg/lane) were resolved on 10% SDS-PAGE followed by Western blotting with an antibody specific to Bcl-2. ***B***. Cells were treated for 24 hrs or 72 hrs with the indicated concentrations of manuka (0.6%–5.0%) or taxol (50 ng/ml). Whole cell extracts (100 µg/lane) were resolved on 10% SDS-PAGE followed by Western blotting with a PARP-specific antibody. The full-length (116 kD) and cleaved (89 kD) forms of PARP are indicated. The cell extracts were also probed with an antibody against β-actin as a control for loading. ***C***. Following treatment for 72 hrs, cells were lysed and DNA extracted, as described in Materials and Methods. Extracted DNA was resolved on 1.5% agarose gel and stained with ethidium bromide to visualize the oligonucleosomal fragments. The results are representati**v**e of two independent experiments.

Bcl-2 is a member of a large family of cell survival-regulating proteins consisting of both pro- and anti-apoptotic regulators [25]. Bcl-2 is a pro-survival protein that acts upstream of the caspase pathway and, when overexpressed, can block cell apoptosis. Conversely, inhibition of Bcl-2 protein expression predisposes to apoptosis. We therefore determined the level of Bcl-2 expression in B16.F1 cells following treatment with manuka or taxol. The results, shown in Figure 5A, demonstrate decreased levels of Bcl-2 protein in manuka-treated cancer cells. In taxol-treated cells, Bcl-2 expression was substantially decreased by 24 hrs of culture and was undetectable by 72 hrs. By contrast, in maunka-treated cancer cells, no decrease in Bcl-2 expression was observed at 24 hrs; however, by 72 hrs, there was >50% reduction in Bcl-2 levels.

One of the target proteins for active caspase-3 is the DNA repair enzyme poly(ADP-ribose) polymerase (or PARP). So, we investigated the effect of manuka treatment on caspase-3 activation by Western blot analysis using a monoclonal antibody against PARP that detects the full length (116 kD) and the cleaved (89 kD) forms of PARP (Fig. 5B). Lysates of B16.F1 cells were prepared following treatment with manuka or taxol for 24 hrs (upper panels) or 72 hrs (lower panels) and subjected to immunoblot analysis with a PARP-specific antibody. After 24 hrs of culture, cleavage of PARP into the 89 kD fragment was evident only in taxol-treated cells (upper panel). However, after 72 hrs, PARP was cleaved effectively in manuka-treated cells in a dose-dependent manner (lower panel). Thus, at concentrations as low as 0.6%, manuka can effectively induce the caspase pathway leading to apoptosis of cancer cells.

The effect of manuka-induced caspase activation on DNA fragmentation was also analyzed by agarose gel electrophoresis of cellular DNA isolated after treatment. As shown in Figure 5C, a characteristic ladder pattern representing fragmented DNA was observed in cancer cells following treatment with manuka. At the highest manuka concentration used (5.0%), the extent of DNA fragmentation, a classical apoptotic feature, was largely equivalent to that observed in taxol-treated cells. Taken together, the above results suggest that manuka leads to inhibition of cellular proliferation through a reduction in pro-survival protein expression and activation of apoptosis pathway.

### In vivo toxicity studies

**Figure 6:**
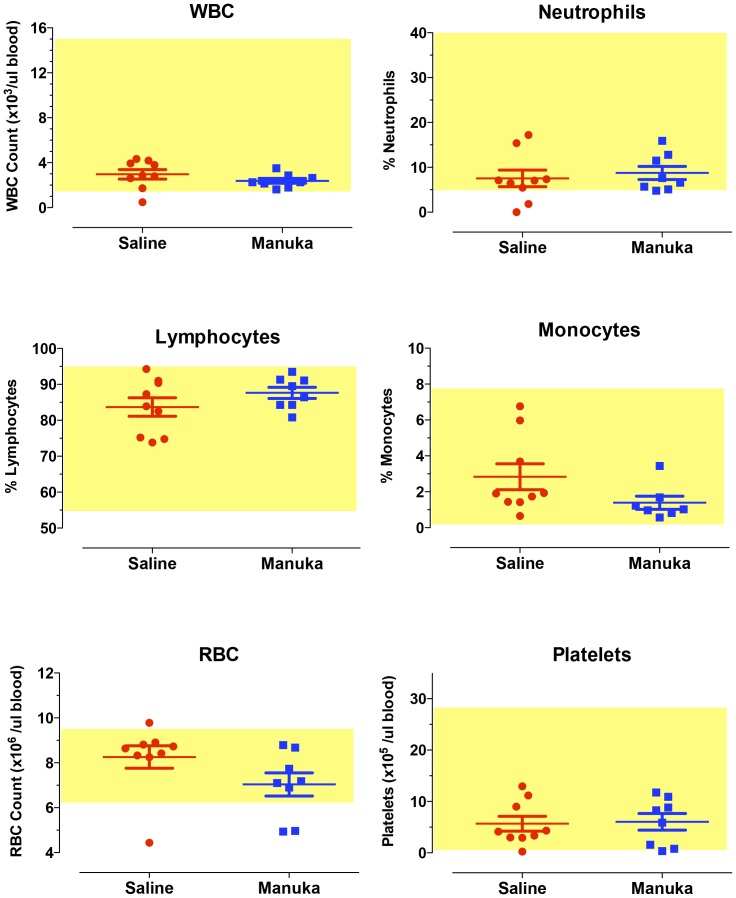
Systemic administration of manuka honey is not associated with any alterations in hematological values. Mice were injected with saline or manuka (50% w/v) 2 times per week for a total of 3 weeks, following which blood was collected and analyzed for the indicated parameters. In each graph, the values for individual mice in a group are shown, together with the mean ± SEM. The shaded box in each graph represents the normal range for that particular parameter. The results are representative of three independent experiments.

**Figure 7:**
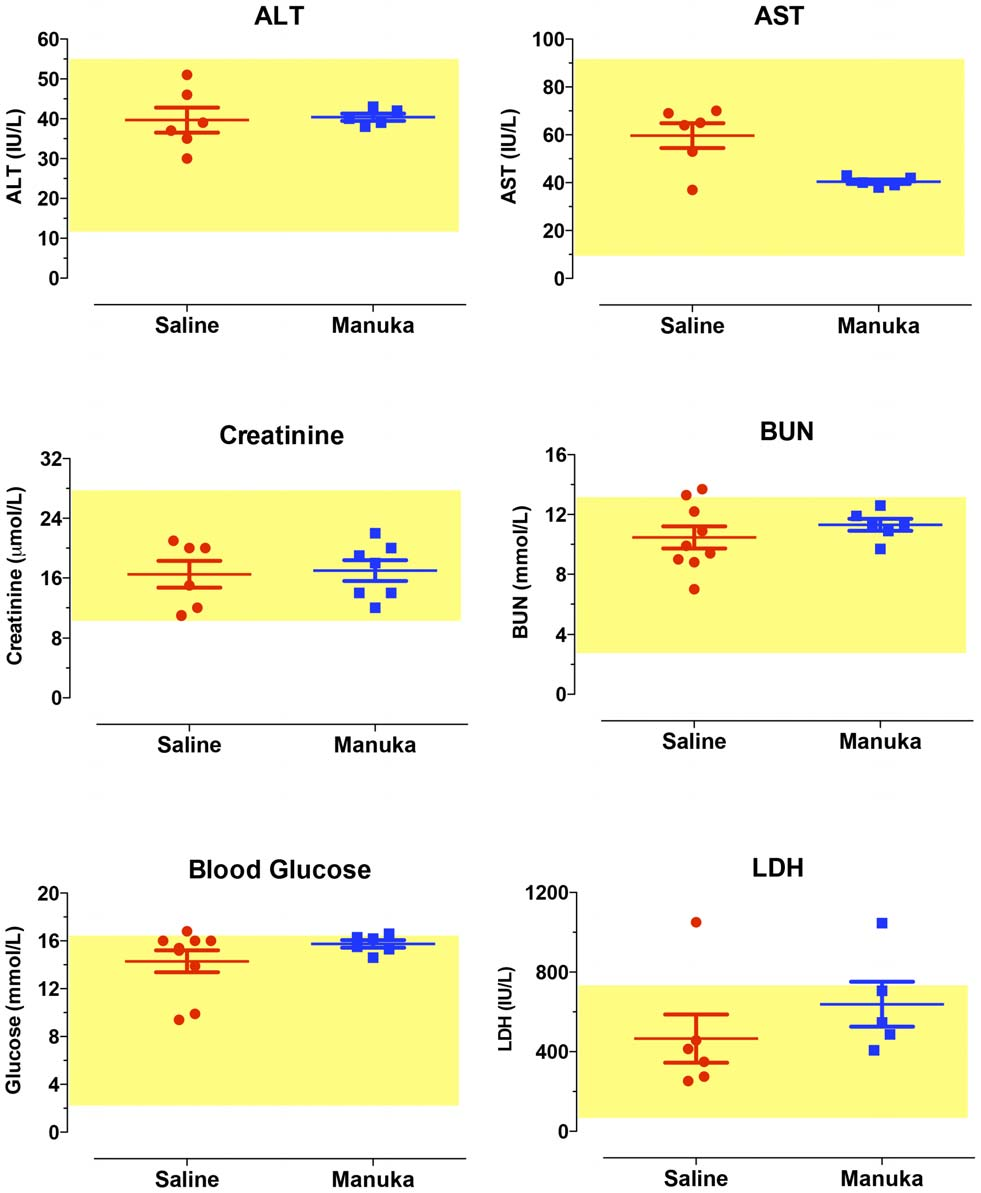
Clinical chemistry parameters are unaltered in mice following intravenous injection with manuka honey. Mice were treated as described in Figure 4 legend, following which blood was collected and analyzed for the indicated parameters. In each graph, the values for individual mice in a group are shown, together with the mean ± SEM. The shaded box in each graph represents the normal range for that particular parameter. The results are representative of three independent experiments.

Given the demonstrated *in vitro* effect of manuka on melanoma cells, we investigated the potential of using manuka in an *in vivo* animal tumor model. In preparation for that, we carried out a series of experiments to test for any potential *in vivo* toxicity associated with intravenous administration of manuka. Mice received multiple i.v. injections of 50% manuka solution diluted in sterile saline for 3 weeks. At the end of this period, animals were sacrificed and blood was collected for hematological and clinical chemistry analysis, the results of which are shown in Figures 6 and 7, respectively. Our findings demonstrated that multiple i.v. injections of manuka were not associated with any alterations in cellular constituents of blood, including total WBC count, RBC count, platelet count, % neutrophils, % lymphocytes and % monocytes (Fig. 6). Furthermore, no significant changes were observed in the levels of various chemical markers of organ dysfunction, including creatinine, BUN, AST, ALT, LDH, and glucose (Fig. 7).

### Effect of manuka on tumor growth in vivo

**Figure 8:**
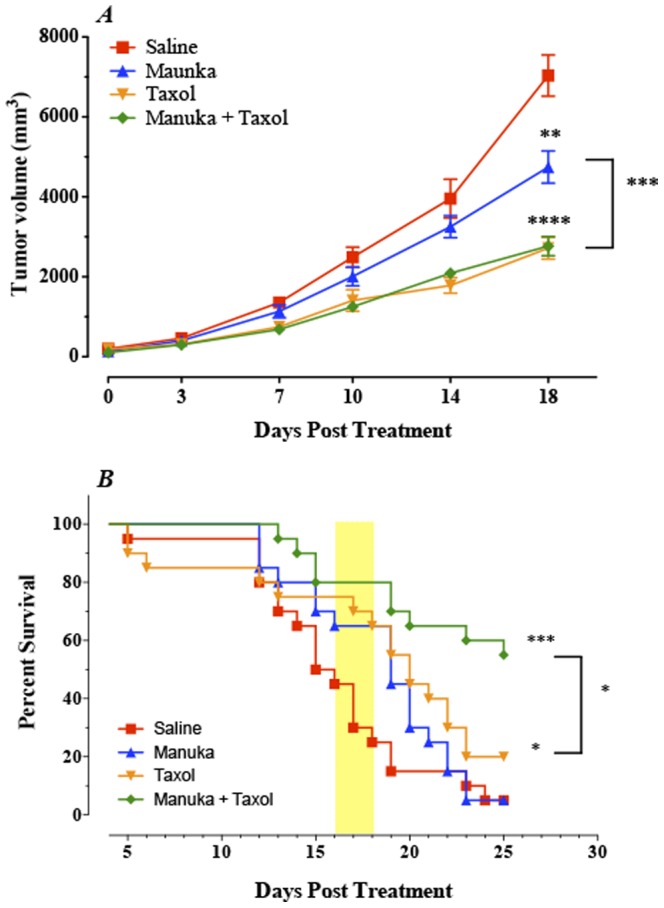
Effect of systemic administration of manuka on tumor growth and host survival. (***A***) Animals with established tumors were treated i.v. with either manuka honey (50% w/v), taxol (10 mg/Kg), manuka+taxol, or saline as control. All treatments were given twice per week until the end of observation period. Each data point represents the mean ± SEM of 19–20 mice per group, pooled from 2 individual experiments. Asterisks denote statistically significant differences between each experimental group and the saline control group; also shown is a comparison between manuka alone and manuka+taxol groups (**, *p*<0.01; ***, *p*<0.001). (***B***) Co-treatment with taxol and manuka leads to a significant enhancement in host survival. Experimental animals were followed for survival for up to day 25 post treatment. Each data point represents the mean ± SEM of 19–20 mice per group, pooled from 2 individual experiments. Asterisks denote statistically significant differences between experimental and saline control groups; also shown is a comparison between taxol alone and manuka+taxol groups (**, *p*<0.01; *, *p*<0.05).

The antitumor activity of manuka was evaluated in the syngeneic B16.F1 melanoma tumor model. C57BL/6 mice with established tumors (mean >50 mm^3^) were divided into four groups and treated by intravenous administration (2 times per week for up to 3 weeks) of manuka alone, taxol alone, manuka plus taxol or saline as control. Tumor volume and animal survival were followed for up to 3 weeks post treatment initiation. As can be seen in Figure 8A, tumor growth in saline-treated mice occurred continuously and rapidly, reaching a mean of 7035±516 mm^3^ by day 18 post treatment, which corresponds to day 31 post tumor implantation. Mice treated with manuka alone exhibited a significant reduction in tumor volume, with a mean of 4744±403 mm^3^, representing ∼33% inhibition of tumor growth (*p* = 0.0029). Mice treated with taxol alone or manuka plus taxol exhibited significantly greater degree of inhibition in tumor growth, with mean tumor volumes being decreased by ∼61% compared to control (*p* = <0.0001). Inhibition of tumor growth in taxol-treated animals was observed as early as 7 days after initiation of treatment, whereas manuka-treated mice exhibited a delay in tumor growth starting on day 10 post treatment (Fig. 8A).

The effect of the various treatments on animal survival was also followed (Fig. 8B). Median survival for saline control group was ∼15 days and great majority of mice (>80%) died by day 19 post treatment. In contrast, manuka-treated mice exhibited enhanced survival initially (shaded box in Fig. 8B) with an overall median survival of 19 days. By ∼3 weeks, however, their survival declined rapidly, and was ultimately comparable to saline controls at the end of the observation period (day 25 post treatment). Similarly, Taxol-treated animals exhibited better survival initially (median survival = 20 days) but then declined reaching an overall survival of 20% at the end observation period. Lastly, mice co-treated with manuka plus taxol exhibited a marked enhancement in their overall survival with 55% of mice surviving (median >25 days), which was significantly different from controls (p = <0.0001). Taken together, these findings demonstrate that intravenously-administered manuka has a modest, but significant, inhibitory effect on the growth of the highly tumorigenic B16.F1 melanoma cells with a transient improvement in host survival. Moreover, when given in conjunction with an optimal dose of taxol, no additive or synergistic effect of manuka on overall tumor volume was observed. However, the combination treatment improved overall animal survival dramatically, suggesting perhaps a role for manuka in reducing drug-induced toxicity.

**Figure 9:**
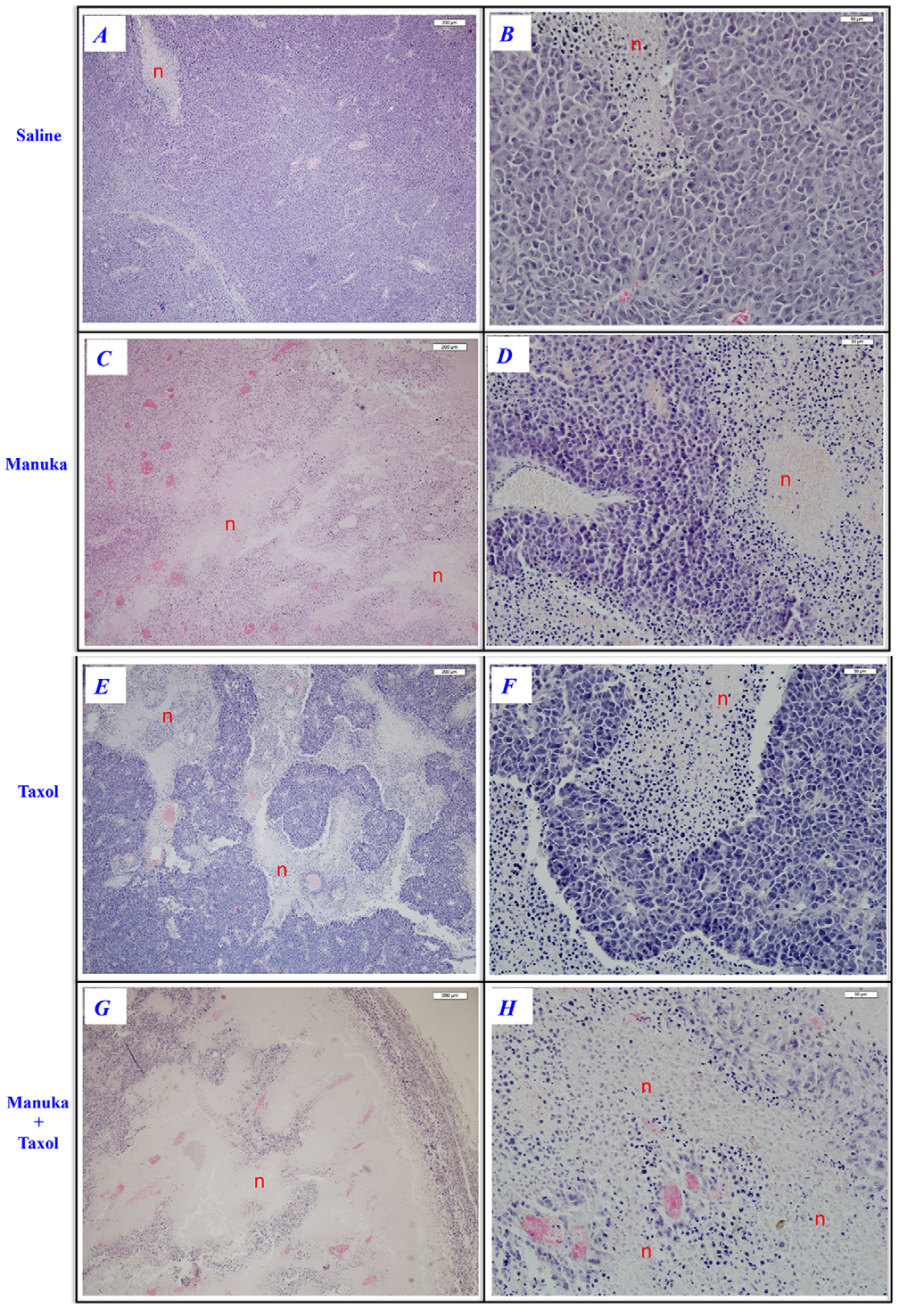
Extent of tumor necrosis in experimental groups following various treatments. Tumors were excised from animals at day 20–24 post treatment with saline (panels ***A–B***), manuka honey (panels ***C–D***), taxol (panels ***E–F***) or manuka+taxol (panels ***G–H***). Tissue sections were stained with H&E, as described in Materials and Methods. For each treatment, representative images at low (panels ***A***, ***C***, ***E***, ***G***; bar = 200 µm) and high magnifications (panels ***B***, ***D***, ***F***, ***H***; bar = 50 µm) are shown. Necrotic regions are indicated (n). The results are representative of two independent experiments.

**Figure 10:**
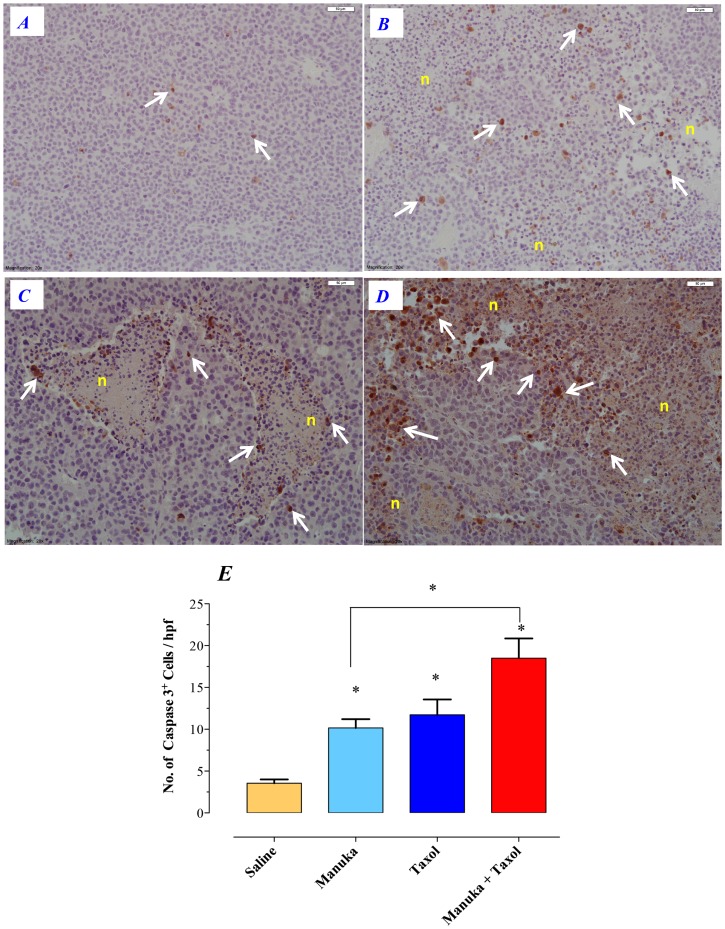
Immunohistochemical staining for intratumor caspase-3^+^ apoptotic cells. Tumor tissue sections were prepared after treatment with saline (panel ***A***), manuka honey (panel ***B***), taxol (panel ***C***) or manuka+taxol (panel ***D***) and stained using caspase 3-specific antibody, as described in Materials and Methods. Representative images at high magnification (bar = 50 µm) are shown. Arrows indicate representative, brown-staining, apoptotic cells. Necrotic regions are also indicated (n). The results are representative of two independent experiments. (***E***) Quantitative estimation of the number of caspase-3 positive cells in tumor sections of different treatment groups. The data is shown as the mean ± SEM of the number of positive cells per high power field. Tumors were obtained from 2–3 mice per treatment group and multiple sections were made from each tumor tissue. The number of positive cells was determined by counting the number of cells in 20 high power fields per section. Asterisks denote statistically significant differences between each experimental group and the saline control group; also shown is a comparison between manuka alone and manuka+taxol groups (*, *p*<0.05).

Tumors excised from animals of various treatment groups were subjected to histological examination with hematoxylin/eosin (H&E) staining as well as immunohistochemical staining for caspase-3. The results of H&E staining are shown in Figure 9. For each treatment group, representative low and high power images are shown, as indicated. In contrast to saline controls (Fig. 9, panels A–B), treatment with manuka alone was associated with the appearance of multiple areas of necrosis within the tumor tissue (panels *C–D*). However, tumors of mice treated with taxol (panels *E–F*) or taxol plus manuka (panels *G–H*) exhibited more extensive areas of necrosis that were intermixed with areas of viable tumor cells. Staining with caspase 3-specific mAb revealed the presence of apoptotic cells, largely concentrated around the perimeter of necrotic tissue (Fig. 10A–D). By counting the number of caspase 3-positive cells in a random selection of 10–20 high power fields (hpf), a quantitative estimate of apoptotic cell number could be achieved. As summarized in Figure 10E, the number of apoptotic cells in tumors of untreated mice was 3.6±0.4 per hpf. In mice treated with manuka or taxol alone, the number of caspase 3-positive cells increased to 10.1±1.0 or 11.7±1.8 per hpf, respectively. In contrast, there was a further increase in the number of apoptotic cells observed in mice treated with taxol plus manuka, reaching a meanof 18.5±2.3 per hpf.

## Discussion

Despite the remarkable advances made over the past 50 years in understanding the basis of cancer development, and the increased availability of treatment modalities, cancer-related death toll remains one of the highest among chronic human diseases. A major concern for anti-cancer drugs is their potential toxicity. Considerable efforts continue to be exerted to identify naturally occurring compounds, or their principle active components, with potential to complement existing cancer therapeutic modalities. The current study highlights several novel findings regarding the utility of manuka honey as a potential anti-cancer agent. First, multiple intravenous injections of manuka, administered over a period of 2–3 weeks, caused no apparent systemic side effects, as judged by the results of the hematological and clinical chemistry analyses which showed no alterations in the cellular constituents of blood or chemical markers of organ dysfunction in the serum of treated animals. Second, manuka treatment resulted in a significant growth inhibition (∼33%) in a melanoma tumor model known for its aggressiveness and low immunogenicity. Third, several lines of *in vitro* evidence demonstrate that manuka induces death of cancer cells via the activation of caspase-9-dependent intrinsic apoptosis pathway. Finally, intravenous co-administration of taxol and manuka resulted in a highly significant inhibition of tumor growth and improved overall animal survival.

Honey has been recognized by many ancient cultures for its healing properties, giving rise to the field of apitherapy. In addition to its recognized antimicrobial and wound healing properties [13], honey has been recently investigated as anti-cancer agent. In an early study, honey was shown to exhibit modest anti-tumor, but good anti-metastatic, activities against a number of tumor cell lines [26]. Another study extended this observation and showed that dietary intake of caffeic acid esters, a major constituent of *Propolis* honey beehives, inhibited the incidence and multiplicity of invasive and noninvasive carcinogen-induced colon adenocarcinomas [27]. More recently, diluted unfractionated honey was shown to inhibit the proliferation of bladder cancer cell lines *in vitro* [17]. Moreover, intralesional injection of honey was found to inhibit tumor growth in a bladder cancer implantation mouse model, but the effect of the treatment on animal survival was not reported [17]. In the present study, we used a melanoma murine model known for its low immunogenicity, and hence high tumorigenicity, to demonstrate a role for manuka honey in retarding tumor growth *in vivo*. Histological and immunohistochemical evidence is provided to show that tumor retardation correlated with increased apoptosis of tumor cells. More intriguingly, our findings demonstrate that simultaneous treatment with a chemotherapeutic drug plus manuka led to a highly significant improvement in overall animal survival. This suggests that the advantage of using intravenous manuka may well extend beyond its direct antitumor activity to include the added beneficial effect of reducing chemotherapy drug-induced toxicity and enhancing host survival.

The main mechanism by which manuka appears to exert its anti-proliferative effect on cancer cells is through the activation of the intrinsic apoptotic pathway, involving the induction of the initiator caspase-9 which in turns activates the executioner caspase-3 [28]. In contrast, no evidence for the activation of caspase-8, and hence the extrinsic pathway, in manuka-treated cancer cells. In contrast, essentially the reverse was observed in taxol-treated cells where caspase-8, but not caspase-9, activation was evident. This is in line with previous reports showing that taxol's effect on cell growth was mediated mainly through the extrinsic apoptosis pathway without the involvement of caspase-9 [24]. Manuka-induced apoptosis is also associated with the activation of PARP, induction of DNA fragmentation and loss of Bcl-2 expression. The results of the *in vitro* cell viability studies demonstrate that manuka was effective against several types of murine and human cancer cell lines at very low concentrations. The IC_50_ values (manuka concentrations required for 50% inhibition of cell growth) of the murine B16.F1 melanoma cells, calculated after 24, 48 or 72 hrs of exposure to manuka honey were 2%, 1.3% and 0.8%, respectively. Similarly, for CT26 cells, the IC_50_ values at 24 and 72 hrs were 2% and 1%. Interestingly, the observed IC50 values for MCF-7 cells are significantly higher, calculated to be >5% and 4% manuka at 24 and 72 hrs, respectively. The observed relative resistance of the MCF-7 cells to manuka-induced apoptosis may well be due to the fact that these cells are known to be deficient in caspase-3 expression [29], [30]. Nevertheless, it is intriguing to hypothesize that the fact that manuka could still induce apoptosis in caspase-3-deficient cells may well indicate that a secondary pathway could also function, albeit at reduced efficiency.

Our findings document the ability of manuka honey, at concentrations as little as 0.3–0.6%, to induce apoptosis in cancer cells. This was demonstrated using several approaches, including cell viability and flowcytometric assays, direct determination of increased caspase 3/7 and 9 enzyme activities, and DNA fragmentation. Moreover, using a syngeneic melanoma model, we could demonstrate that manuka was also effective against cancer cells *in vivo*, as evidenced by the observed decrease in tumor volume and increased apoptosis of tumor cells detected by caspase-3 immunohistochemical analysis. Although detailed analyses of the effect of other types of honey on cancer cells remain to be done, based on the cell viability data, our results suggest that manuka honey may be superior in its anticancer potential than other types of honey. Using Tualang honey, Ghashm and coworkers reported IC_50_ values of 3.5–4.0% against human oral squamous cell carcinoma and osteosarcoma cell lines [16]. Swellam et al also reported IC_50_ values of 2.0–4.0% against bladder cancer cell lines using unfractionated honey from Manitoba, Japan [17]. These differences are most likely due to variations in honey content, particularly in polyphenols and phenolic acids with known antitumor activities [15].

Scientific evidence for the use of honey in wound healing has been accumulating over the past few years, largely as a result of completed small-scale clinical trials [31], [32]. Many properties of honey have been described that aid the process of wound healing such as activating the innate immune system, inducing the migration of neutrophils and macrophages, promoting the debridement of devitalized tissue, stimulating angiogenesis and granulation, and preventing infection [13], [33]. Manuka honey has the capacity to stimulate macrophages to release innate immune mediators, such as TNF-α, IL-1ß and IL-6, which are essential for tissue healing and for limiting microbial infections [2], [11].

An intriguing finding of the present study is the beneficial effect of administering manuka together with taxol. In comparison with the animal group receiving taxol alone, those treated with taxol and manuka exhibited a highly significant improvement in survival. This occurred despite having almost identical mean tumor volumes in both experimental groups, suggesting there was no added or synergistic action of both agents on inhibiting tumor growth, at least at the optimal dose of taxol used in this study. These findings lead us to hypothesize that manuka administration may decrease the toxic side effects of chemotherapeutic drugs. Support for this hypothesis is evident in recently published reports demonstrating potent anti-inflammatory, antioxidant and cell growth-promoting activities among various types of honey, including manuka [8], [34]. Moreover, intravenous administration of honey protected against organ failure in rabbits following LPS-induced sepsis through the inhibition of inflammation and myeloperoxidase production [35]. Thus, manuka honey may well improve survival of taxol-treated, tumor-bearing, mice via a similar protective mechanism. The current findings should facilitate further work to examine whether manuka could synergize with, or be a substitute for, chemotherapeutic drugs given at sub-optimal doses for cancer therapy.
